# Supplementary material for: Improved visual detection of DNA amplification using pyridylazophenol metal sensing dyes
Source: Commun Biol. 2022 Sep 21;5:999. doi: 10.1038/s42003-022-03973-x (PMC9491268; doi:10.1038/s42003-022-03973-x)

SUPPLEMENTARY MATERIAL FOR:

Improved Visual Detection of DNA Amplification Using Pyridylazophenol Metal Sensing Dyes

Yinhua Zhang, Eric A. Hunt, Esta Tamanaha, Ivan R. Corrêa, Jr., and Nathan A. Tanner

Supplementary Figure 1. Only Mn^{2+} is capable of promoting PAPS dyes color change in response to LAMP amplification. Metal ions were assayed at 100 µM concentration with 75 µM 5-Bromo-PAPS or 5-Nitro-PAPS in triplicates, with 1 ng Lambda DNA (1 ng) or without (NTC) and the reaction color in the presence of each metal ion is shown for before (Left column) and after (Middle column) LAMP amplification. The reactions with Mn^{2+} are highlighted with dashed rectangles. Representative real-time curves for some metal ions were shown in the right column. a) Bromo-PAPS, b) Nitro-PAPS.

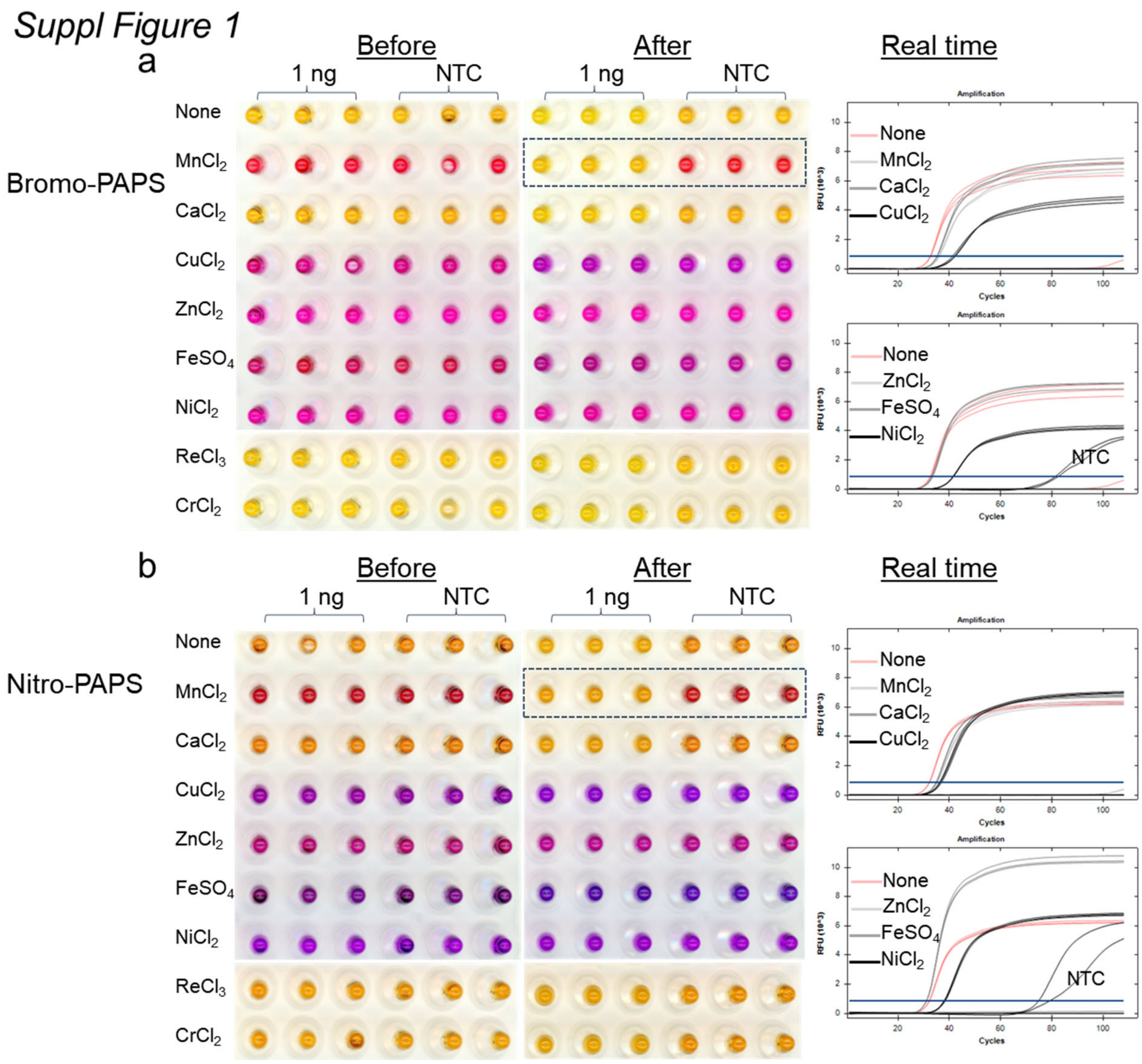

Supplementary Figure 2. Time course of LAMP reaction color change. Replicate LAMP reactions were set up in 6 PCR strip tubes using E1700 or E1708 LAMP mixes with 1 ng lambda target or NTC in duplicate and incubated on a Bio-Rad CFX real-time instrument monitored via the LAMP Fluorescent dye. 1 strip was removed at each indicated timepoint (0, 8, 16, 24, 32, and 40 minutes) and kept at room temperature. At the end of the last time point, the reaction color was scanned and absorbance at 450 nm and 550 nM recorded for all strips. a) Visual color of reactions at different time point aligned with corresponding real time curves (jagged vertical line indicates the instrument lid was opened during a plate read). b) Absorbance changes of between 450 nm and 550 nM plotted over incubation time. Visual color was starting to change at 16 minute time point, with fluorescent curve rising slightly faster than the visual/absorbance signal, and color change was clear by 24 minutes.

Suppl Figure 2

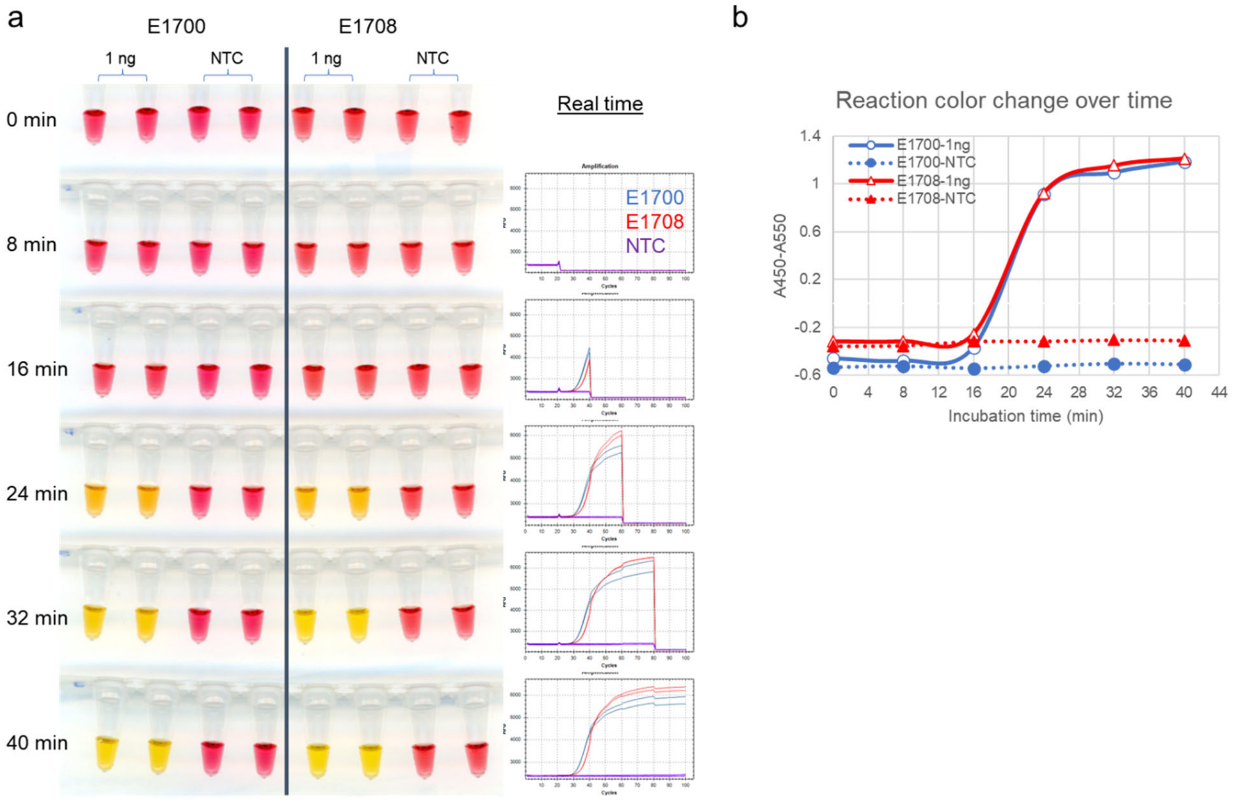

Supplementary Figure 3 Detection of LAMP amplification with a PAPS dye tolerates more carryover solutions. The effectiveness of visual detection is shown for 5-Bromo-PAPS (PAPS) and a pH-based indicator (phenol red) in the presence of increasing amounts of carryover solution. The reactions boxed with dotted rectangles indicate reactions that tolerated the highest amount of carryover solutions. Real-time curves (with color coding for different amounts of carryover solution) for LAMP reactions are presented at the bottom. a, Effect of the elution buffer (EB). b, Effect of the viral transport medium (VTM).

Suppl Figure 3

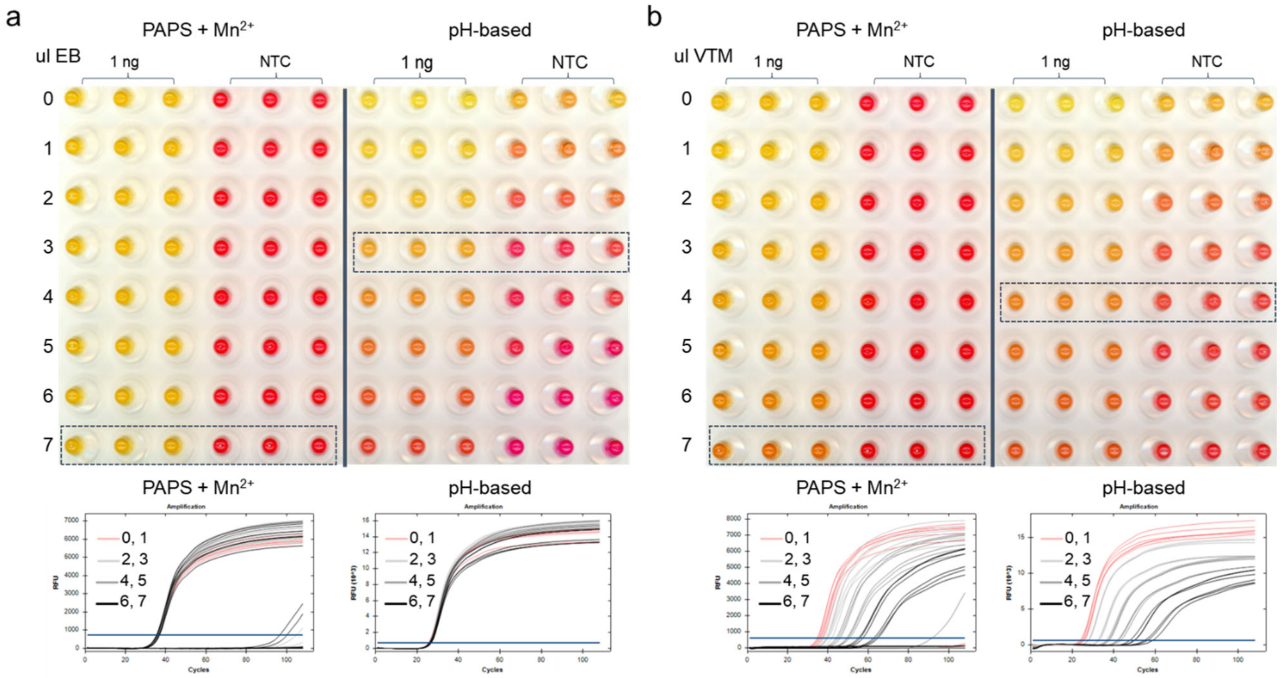

Supplementary Figure 4. Effect of EDTA on LAMP reaction color. LAMP reactions were performed in the presence of 100 uM 5-Bromo-PAPS and 0-400 uM of EDTA with Mn^{2+} at either 75 µM or 175 µM. The reaction plate was scanned before incubation and after incubation, and the amplification was also monitored using real time fluorescence. a) Reactions with 75 µM Mn^{2+}. Color change between the reactions with target and NTC started to show less contrast when the concentration of EDTA increased above 75 µM. b) Reactions with 175 µM Mn^{2+}. Robust color change was observed even in the presence of 100 µM EDTA between the reactions with target and NTC. When EDTA increased above 200 µM, reactions with either 75 µM Mn^{2+} or 175 µM Mn^{2+} had yellow color before amplification and no color change was possible. In all conditions LAMP amplification was not suppressed by these EDTA concentrations as shown in the real time curves.

Suppl Figure 4

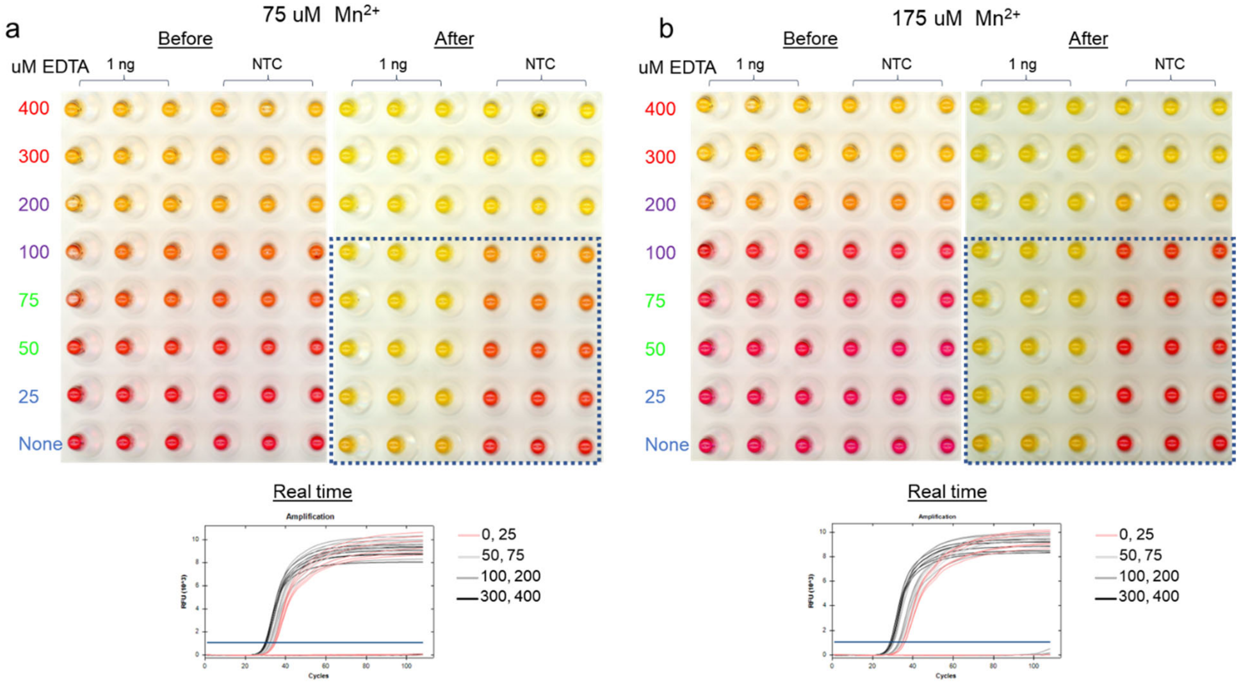

Supplementary Figure 5. Time course of color change and DNA yield in PCR reactions with Lambda amplicons. Triplicate PCR reactions are shown with amplicons of 0.5 kb. 1.0 kb and 2.0 kb with 1 ng lambda DNA (~1.91x10^7 copies) and NTC control reactions with primers for the 0.5 kb amplicon but without template DNA. Reactions underwent 16, 20, 24, 28 32 and 36 PCR cycles. a) Visual color change of PCR reactions. b) Absorption gain at A450 nm vs A550 nm plotted against PCR cycle numbers. An arbitrary threshold (dashed line) of 0.10 was drawn in the graph for visible color change perceivable by the naked eye. c) DNA yield in reactions was measured and plotted over PCR cycles . The visual color change threshold (dashed line) corresponds to ~ 60 ng/μL DNA yield in the PCR reactions.

Suppl Figure 5

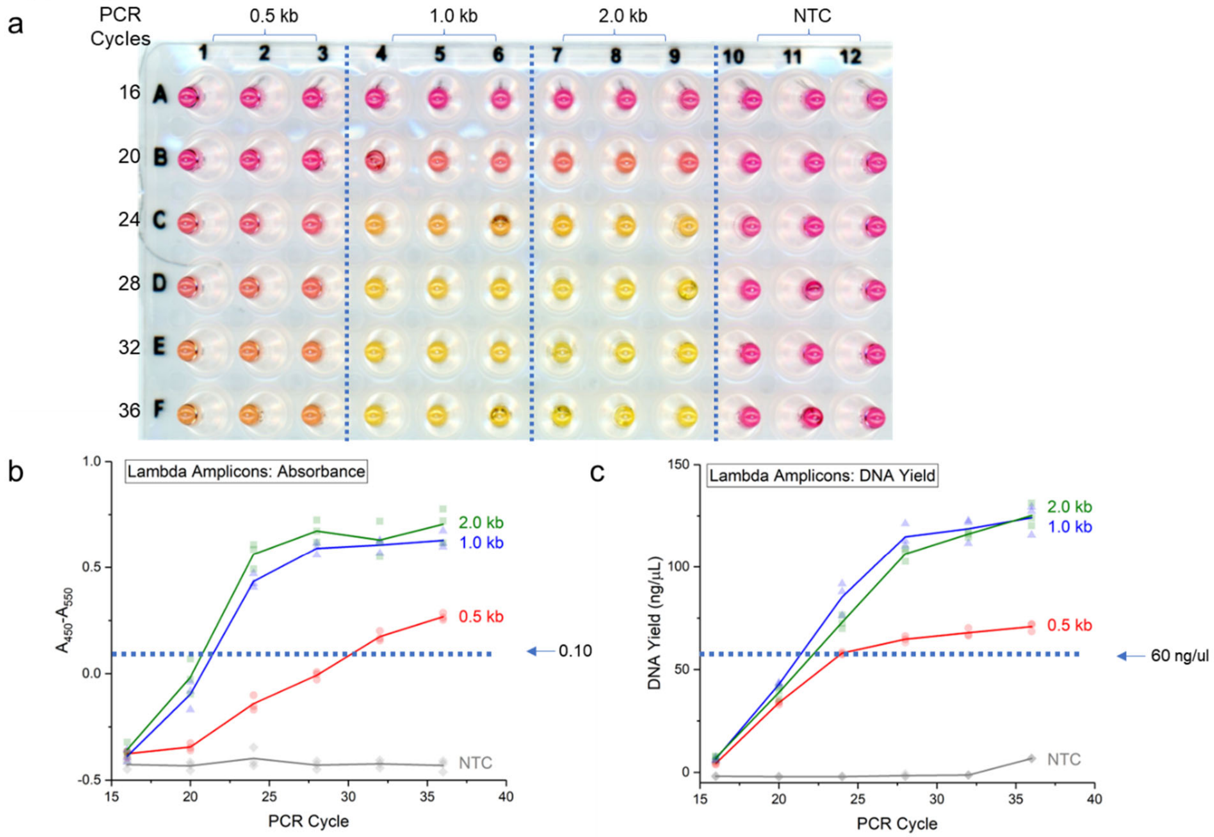

Supplementary Figure 6. Time course of color change and DNA yield in PCR reactions with human amplicons. Triplicate PCR reactions are shown with human amplicons of 0.5 kb. 1.0 kb and 2.0 kb with 10 ng of human genomic DNA (~2,900 copies). PCR reactions underwent more PCR cycles (24, 28, 32, 36, 40 and 44 cycles) than that with lambda amplicons due to much reduced template copy numbers. a) Visual color change of PCR reactions. b) Absorption gain at A450 nm vs A550 nm plotted against PCR cycle numbers. An arbitrary threshold (dashed line) of 0.10 was drawn in the graph for visible color change perceivable by the naked eye. c) DNA yield in reactions was measured and plotted over PCR cycles. The visual color change threshold (dashed line) corresponds to ~ 50 ng/µL DNA yield in the PCR reactions, which is slightly less than that of lambda amplicons.

Suppl Figure 6

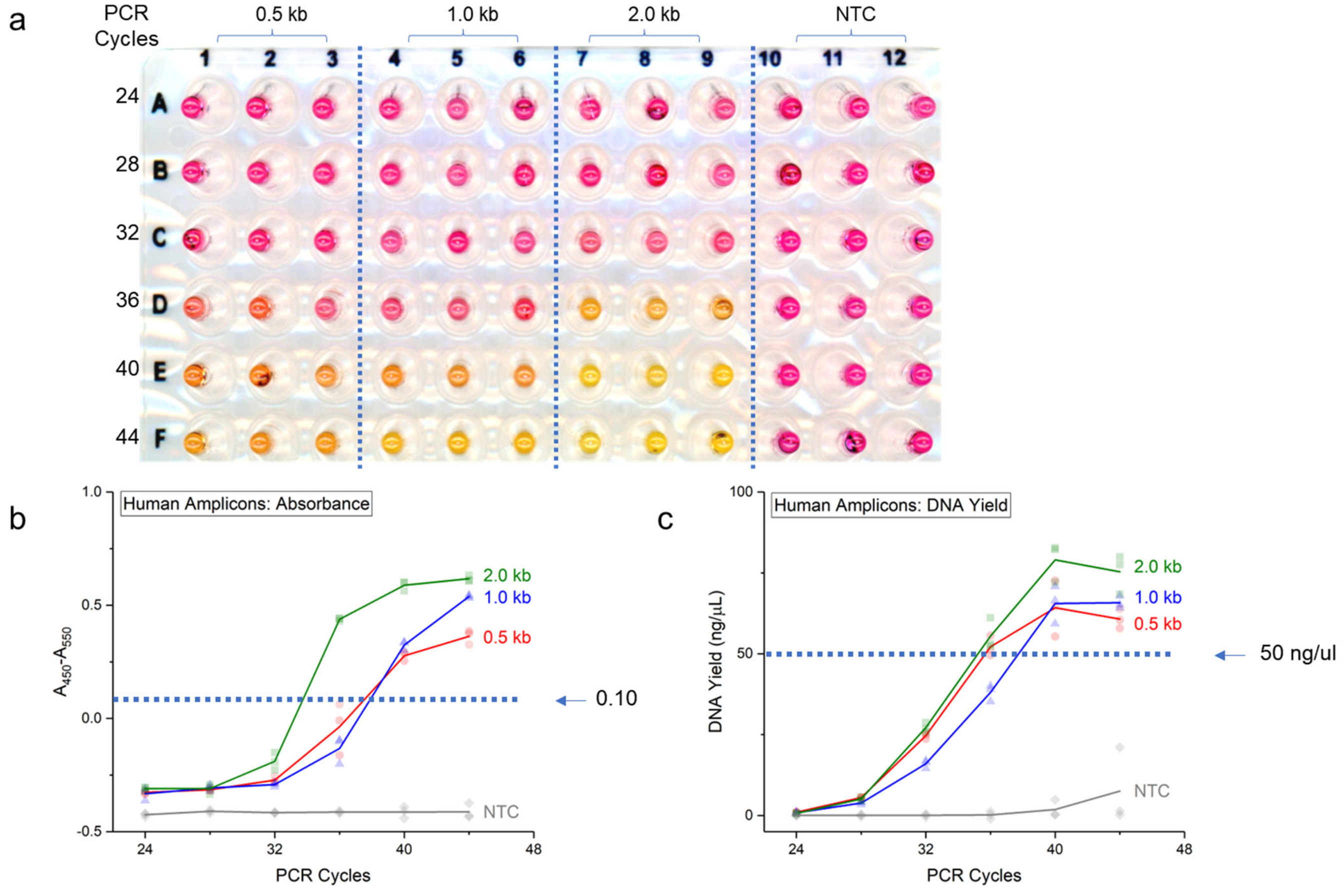

Supplementary Figure 7. Correlation of color change with DNA yield in PCR reactions.

The average absorption gains (A450-A550) (Y-axis) of PCR reactions were plotted against DNA yield (X-axis) for lambda and human amplicons along 5 sampling points of PCR reactions (16, 20, 24, 28 32 and 36 cycles for lambda amplicons and 24, 28, 32, 36, 40 and 44 cycles for human amplicons). The horizontal red dashed line corresponding to A450-A550 of 0.10, which is a point where a color change is perceivable by the naked eye. The DNA concentrations intercepted with this threshold were obtained as the DNA yield required to see color change and depicted in Figure 6d. a) Lambda amplicons. b) Human amplicons.

Suppl Figure 7

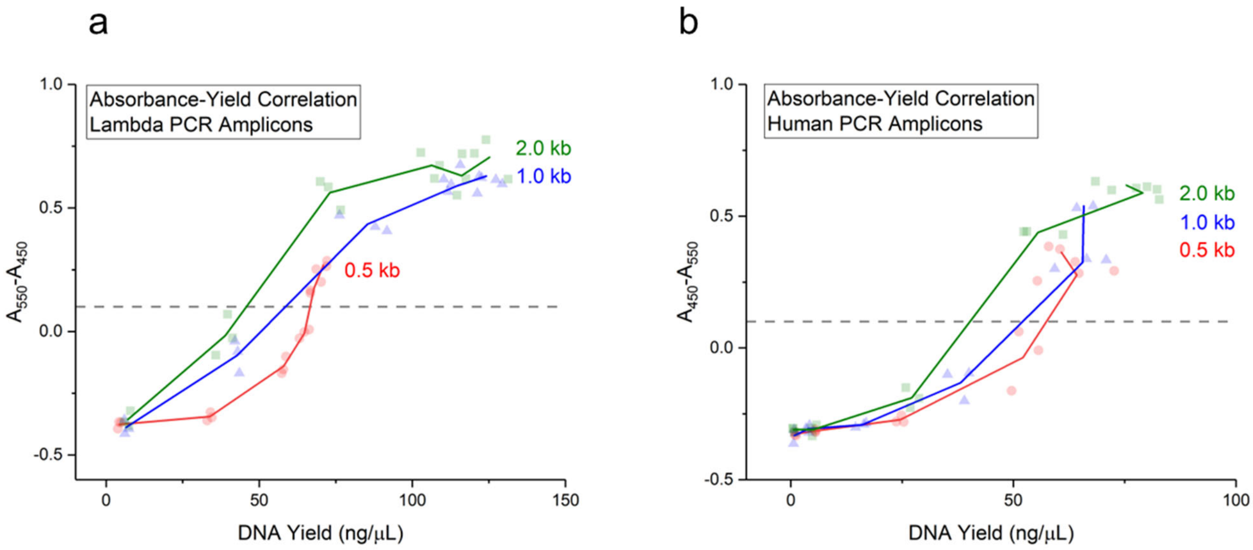

Supplement: Supplementary file 2 — Supplementary Information [file 42003_2022_3973_MOESM2_ESM.pdf]
