# Supplementary material for: Improved visual detection of DNA amplification using pyridylazophenol metal sensing dyes
Source: Commun Biol. 2022 Sep 21;5:999. doi: 10.1038/s42003-022-03973-x (PMC9491268; doi:10.1038/s42003-022-03973-x)
Supplement: Supplementary file 3 — Description of Additional Supplementary Files [file 42003_2022_3973_MOESM3_ESM.pdf]

Description of Additional Supplementary Files

File name: Supplementary Data

Description: The source data behind Supplementary Figures 5b, c, 6b, c, and 7
